# Urinary Hydration Biomarkers and Water Sources in Older Adults with Neurocognitive Disorder

**DOI:** 10.3390/nu15030548

**Published:** 2023-01-20

**Authors:** Cátia Queirós, Flávia Borges Machado, Duarte Barros, Joana Sampaio, Arnaldina Sampaio, Renata Barros, Pedro Moreira, Óscar Ribeiro, Joana Carvalho, Patrícia Padrão

**Affiliations:** 1 Faculdade de Ciências da Nutrição e Alimentação da Universidade do Porto, 4150-180 Porto, Portugal; 2 Faculdade de Desporto da Universidade do Porto, 4200-450 Porto, Portugal; 3 Centro de Investigação em Atividade Física, Saúde e Lazer, Universidade do Porto, 4200-450 Porto, Portugal; 4 Laboratório para a Investigação Integrativa e Translacional em Saúde Populacional (ITR), 4050-600 Porto, Portugal; 5 EPIUnit-Instituto de Saúde Pública, Universidade do Porto, 4050-600 Porto, Portugal; 6 Centro de Investigação em Tecnologias e Saúde (CINTESIS), Departamento de Educação e Psicologia da Universidade de Aveiro, 3810-193 Aveiro, Portugal

**Keywords:** hydration status, urinary osmolality, water sources, older adults, neurocognitive disorder

## Abstract

The risk of dehydration in older adults with neurocognitive disorder (NCD) is controversial. The purpose of this study was to assess hydration status, its determinants, and water intake sources in older adults with NCD. A sample of 30 participants (≥60 years) was included. Sociodemographic, clinical data and one 24-h urine sample were collected. Urinary osmolality, sodium, potassium, volume, and creatinine were quantified. Inadequate hydration status corresponded to urine osmolality > 500 mOsm/Kg, or a negative Free Water Reserve (FWR). Two 24-h food recalls were used to assess dietary intake and water sources. The adequacy of total water intake (TWI) was estimated according to EFSA. The contribution of food and beverages to TWI was calculated, and their associations with the urinary osmolality median were tested. Of the total number of participants, 30% were classified as having inadequate hydration status, with no differences between sexes. Regarding TWI, 68.4% of women and 77.8% of men did not reach the reference values. Water (23%), followed by soup (17%), contributed the most to TWI, while vegetables (2%) and alcoholic/other beverages (3%) contributed the least. According to the median urinary osmolality, there was no significant difference in sociodemographic/clinical characteristics. It is critical not to overlook hydration in this vulnerable population.

## 1. Introduction

Water is the main component of the human body and is essential for life and the maintenance of homeostasis. In older adults, water accounts for approximately 50% of the body weight [1,2,3,4,5,6], and this period is one of the life cycle phases in which the risk of dehydration is most imminent [2,5,7,8,9,10]. Age-related changes may cause an increased risk of dehydration by decreasing the sensitivity to thirst and decreasing the total body water (TBW). In addition, changes in the kidney make it less able to conserve body water.

In addition to all of these physiological changes that drive older adults to a higher risk for dehydration, a frequent loss of autonomy in their daily life [2,5,11,12,13,14,15], together with clinical conditions related to dysphagia or urinary incontinence may affect the fluids ingestion [2,16]. There is a wide range of estimates of the prevalence of dehydration in older adults, between 20% and 80%, depending on the population being evaluated and the methods used [2].

Neurocognitive disorder (NCD) prevalence has increased over time and is estimated to triple in the next 30 years [17,18,19].

There are some dietary factors that may be related to hydration status. An example is ingested sodium, which plays a role in controlling urinary volume and fluid intake; that is, a diet with high levels of sodium can generate an increase in fluid intake to compensate for the salt ingested and, consequently, a greater urine volume (to excrete excess salt). In addition to this, a high sodium and low potassium intake is a well-known risk factor for cerebrovascular disease, which tends to affect the elderly and the elderly with NCD [3,8,20,21,22].

The hydration status is influenced by the loss of water and the water that enters the body. Under physiological conditions, the kidney excretes the lost water, and the water that enters the organism comes from the water in drinks and foods. A small part of the water is generated through oxidation. According to the assumption that the renal system is fundamental to water balance, urinary biomarkers have emerged with special interest [23,24,25,26,27].

The 24-h osmolality has been considered an interesting biomarker of hydration throughout the day because it represents the sum of all behavioral responses and neuroendocrine functions that influence renal concentration or dilution. It is highly responsive to total water intake (TWI) and thus best suited to determine individual water needs [3,10,28].

Urinary biomarkers for detecting dehydration can be influenced by kidney function, and with aging, this is often impaired. This may lead to reluctance to use these biomarkers to assess dehydration in older people [23,24,27] and collecting 24 h urine samples in older adults is challenging [28]. Therefore, there is limited data on this population.

Older adults with NCD are at a higher risk of dehydration because they have cognitive changes that can make communication difficult, as well, as low independence, which often results in the need for dietary assistance [2,29,30].

Thus, the aim of the study was to evaluate the hydration status through urinary biomarkers such as urinary osmolality, its determinants (sociodemographic and clinical), and which foods and beverages contribute most to hydration in an elderly population with NCD.

## 2. Materials and Methods

### 2.1. Population and Study Design

A cross-sectional observational study was conducted in adults with NCD (≥60 years old), in Porto. The participants belong to a sub-sample of the “Body & Brain” study (ClinicalTrials.gov ID: NCT04095962), and the protocol was published elsewhere [31]. The following participants were considered eligible: individuals aged ≥ 60 years; diagnosed individuals with dementia or neurocognitive disorder using accepted diagnostic methods criteria such as those established by the Diagnosis and Statistical Manual of Mental Disorders (DSM-IV-TR or DSM-5) [32], International statistical classification of diseases and related health problems (ICD-10) [33] or National Institute of Neurological and Communicative Disorders and Stroke and the Alzheimer’s Disease and Related Disorders Association (NINCDS-ADRDA) [34] and the diagnostic has been done by a physician for at least 6-months.

It was approved by the Ethical Committee of the Faculty of Sports of the University of Porto (Ref CEFADE22.2018), and the participants and caregivers/legal representatives were asked to sign an informed consent. All procedures performed during the study were in accordance with the 1964 Helsinki Declaration.

From the total number of "Body and Brain" participants (n = 122), those who collected one 24-h urine sample at baseline were considered eligible for the present study (43 participants). The final sample was composed of 30 participants since 13 subjects were excluded due to incomplete urine samples.

### 2.2. Sociodemographic and Clinical Data

To collect information on sociodemographic and clinical data, a structured face-to-face questionnaire was applied to the individuals with NCD and to their caregivers by trained interviewers. Sociodemographic data included sex, age, education, marital status, and type of residence. The level of education corresponded to the number of school years completed, and it was further divided into non-formal education (if not attending school) or formal education (if attending primary, secondary, or higher education). Marital status was categorized as married, in a civil union, single, or widowed. The type of residence was classified as living with family, institutionalized, or alone. Regarding clinical data, information was collected on the aetiological subtypes of NCD, medication intake, and the presence or absence of chronic diseases. All clinical data were self-reported. The data were confirmed with the clinical records of individuals who were institutionalized. In the case of individuals living in the community, the reported information was confirmed with caregivers and family. From the latter, the number of comorbidities was computed.

The severity of NCD was assessed using the Mini-Mental State Examination questionnaire, adapted and translated for the Portuguese population, which was used to map the categories of the Clinical Dementia Rating questionnaire [35].

### 2.3. Urinary Biomarkers and Hydration Status

The interviewers gave participants and/or their caregivers oral and written instructions on how to proceed with the collection and storage of the 24-h urine sample and provided a container to collect urine. A certified laboratory was responsible for urine sample analysis. Through the 24-h urine sample, data on urinary osmolality, sodium and potassium urinary excretion, urinary volume, and urinary creatinine were assessed. A urine sample was considered complete if the creatinine level was >0.4 g/24 h for women and >0.6 g/24 h for men [36] or if the volume collected was >500 mL [28]. Information on the season of urine collection was also gathered.

It was considered an inadequate hydration status if the urinary osmolality was higher than 500 mOsm/kg, which corresponded to an undesirable hydration status according to the European Food Safety Authority (EFSA) [37], or if the Free Water Reserve (FWR) was negative, which represents a risk of hypohydration/ hypohydration according to Manz et al. [38]. Free water reserve (mL/24 h) is calculated by subtracting 24-h urine volume from obligatory urine volume [38].

Free Water reserve=24 h urine volume−obligatory urine volume

where the obligatory urine volume is the ideal urine volume required to excrete the actual 24 h urine solutes and is calculated by the formula:

$$Obligatory\;urine\;volume=\frac{24\;h\;urine\;solutes\;\left(mOsm/d\right)}{\left(830-3.4\times \left(age-20\right)\right)}.$$

To assess the hydration status determinants, the sample was stratified according to the median urinary osmolality, due to the absence of specific osmolality cut-off points in older adults.

Daily excretions of sodium and potassium were converted from mmol to milligrams by multiplying by their atomic weight. Excessive sodium excretion was defined as ≥2000 mg/day; potassium excretion was considered insufficient if <3510 mg/day; and the Na/K molar ratio > 1 was considered excessive, according to the World Health Organization (WHO) cut-offs [39,40].

### 2.4. Anthropometrics Data and Body Composition

Weight was measured using a weighting scale, in kilograms, and height was measured with a stadiometer, in meters. The body mass index was calculated using the formula [body weight (kg)/(standing height x standing height (m))]. Participants were classified according to the WHO cut-off values as underweight (<18.5 kg/m^2^), normal weight (18.5–24.9 kg/m^2^), overweight (25.0–29.9 kg/m^2^), or obese (≥30 kg/m^2^) [41]. Due to the low number of participants in each category, participants were grouped into two categories: “underweight” or “normal weight” and “overweight” or “obesity”.

Waist and hip circumferences were assessed at the midpoint between the iliac crest and the bottom of the ribcage and at the widest portion of the buttocks, with the tape parallel to the floor, respectively, using a tape measure. Participants were classified according to the risk of metabolic complications using the waist circumference: no risk (women: <80 cm; men: <94 cm); high risk (women: ≥80 cm and ≤88 cm; men: ≥94 cm and ≤102 cm); and very high risk (women: >88 cm; men: >102 cm) [42], further grouped into two categories: “no risk” and “high risk/very high risk”. For the waist-hip ratio, the following cut-offs were considered: men ≥0.90 cm and women ≥0.85 cm for substantially increased metabolic risk [43].

Body composition was assessed using dual-energy X-ray absorptiometry (Hologic QDR 4500, Explorer model, version 12.4). The average percentages of fat-free mass and fat mass in relation to the total body mass were obtained.

### 2.5. Instrumental Activities of Daily Living

Older adults with NCD may experience significant declines in one or more cognitive domains, interfering with daily activities. The Barthel Index, adapted and translated for the Portuguese population, was used as an instrument to assess basic activities of daily living (BADL). Each of the 10 BADLs presented considers between two and four levels of dependence, with a score of 0 corresponding to total dependence. In turn, independence is scored with 5, 10, or 15 points, depending on the levels of differentiation. The total score of this index varies between 0 and 100 points, with higher scores indicating a lower degree of dependence.

Scores of 100 to 90, 89 to 60, 55 to 40, 35 to 20 and <20, mean that the individual is independent, slightly dependent, moderately dependent, severely dependent, and totally dependent, respectively [44].

### 2.6. Physical Activity

The Modified Baeck Physical Activity, adapted and translated for the Portuguese population, was applied. The questionnaire consists of eight items grouped into two dimensions: physical activity related to sport and other activities during leisure time. Total physical activity is calculated by adding the values of each dimension. Higher total scores indicated a higher level of physical activity [45].

### 2.7. Quality of Life

Quality-of-Life Alzheimer’s Disease Scale, adapted and translated for the Portuguese population, was used to measure the participants’ quality of life. The questionnaire was applied to the participant and the caregiver, and its final score weights the scores of the two questionnaires, the caregiver’s and the individual’s. The higher the total score, the better the individual’s quality of life [46].

### 2.8. Contribution of Food and Beverages to Total Water Intake

Two 24-h food recalls were applied on non-consecutive days and on days as close as possible to the collection of 24-h urine samples. Individuals with NCD were asked to recall, in detail, all the foods and drinks consumed in the previous 24 h, including the type of food and the portions. This information was confirmed by the caregiver, and if necessary, the caregiver and the individual were consulted simultaneously. For the conversion of food into nutrients, including the contribution of water from foods, the Food Processor Plus^®^ program version SQL 11.11.32 (ESHA Research Inc., Salem, OR, USA) was used. Food and beverage groups were created according to water content (Table 1) to estimate the contribution of food groups to TWI, dietary intake, and their association with urinary osmolality. According to the recommendations of the EFSA, the TWI, considering food and all types of beverages, was considered inadequate if it was <2.5 L/day for men and <2 L/day for women.

## 3. Statistical Analysis

The normality of the variables was tested using the Kolmogorov-Smirnov test. Categorical variables were presented using frequencies. According to the normal distribution of the variables, the results were described by the mean and standard deviation, or by the median and the 25th and 75th percentiles. Descriptive statistics were used to characterize the sample and Student’s *t*-test and Mann’s test—Whitney were used to compare the means, or medians, respectively, of sociodemographic and clinical variables, according to the median of urinary osmolality. Pearson’s chi-square test was applied to determine whether there is a significant difference between the expected and observed frequencies in one or more categories. Results were considered significant when the *p* value < 0.05. Statistical analyses were performed using the Software Package for Social Sciences for Windows (version 26.0, 2021, IBM (SPSS, Inc., an IBM company, Chicago, IL, USA)).

## 4. Results

The characteristics of the final sample are detailed in Table 2. The sample was composed of 30 participants (66.7% female) with a mean age of 76.4 (7.3) years, ranging from 61 to 88 years. Regarding sociodemographic variables, 9 (32.2%) had no formal education, 19 (63.6%) were married or in a civil union, and 23 (76.7%) lived with a family member.

For subtypes of major NCD aetiology, it was observed that 9 (33.3%) of the population had no specified subtype, 5 (18.5%) had NCD due to Alzheimer’s disease and multiple aetiologies, and NCD due to vascular disease was present in 3 (11.1%). Finally, 1 (3.3%) had NCD due to substance/medication induced symptoms (Wernick-Korsakoff syndrome). The relation between the characteristics of the participants and the severity of NCD was also studied (Table A1. Characteristics of the participants according to the severity of major NCD). No significant results were found, except for the number of comorbidities, where the average of comorbidities is higher in the “Questionable NCD–Mild NCD” category (4.8 vs. 3, *p* = 0.02).

Regarding participants’ clinical data, 16 (57.1%) were hypertensive; 19 (67.9%) were using antihypertensive medications; 21 (75%) had dyslipidaemia; 8 (28.6%) diabetes mellitus; and 2 (7.1%) had kidney disease. From the sample, 2 (7.1%) reported to have had myocardial infarction and 5 (17.9%) stroke.

It was observed that 9 (30%) of participants had an inadequate hydration status, where in 9 (30%) had a urinary osmolality > 500 mOsm/kg and 2 (6.7%) had negative FWR.

The median (percentile 25; percentile 75) of urinary osmolality and FWR were 437.5 (376; 529.5) mOsm/kg and 400 (300) mL/24 h, respectively. No significant differences between sexes were observed.

The mean (standard deviation) of the urinary volume was 1271 (349) mL/day. The mean (standard deviation) of urinary sodium excretion was 2457 (953) mg/day; for potassium, it was 1924 (581) mg/day; and for the Na/K molar ratio, it was 2.2 (0.8). Of the total, 21 (70%) participants had an excessive sodium excretion, 29 (96.7%) had an insufficient potassium excretion, and 28 (93.3%) had an excessive Na/K. Considering the season of urine collection, 21 (70%) of participants collected urine in the autumn, 6 (20%) in the spring, 2 (6.7%) in the winter, and 1 (3.3%) in the summer.

Regarding the characteristics of the participants according to the median urinary osmolality, no significant statistical differences were found (Table 2).

The TWI was, on average (standard deviation), 2007 (829) mL, 1936 (839) mL in women, and 2156 (834) mL in men.

No significant statistical differences were found in TWI, water sources, or dietary intake according to the median urinary osmolality (Table 3).

**Figure 1:**
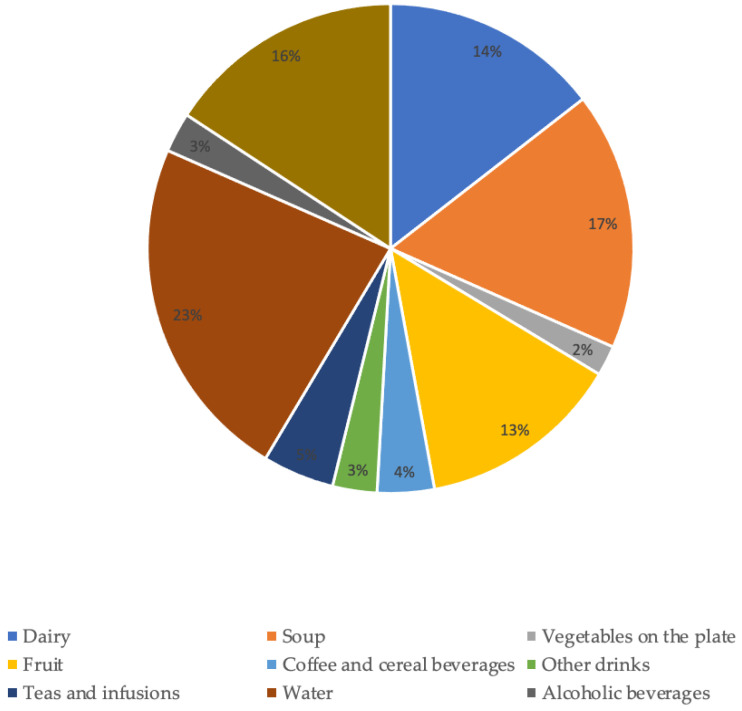
Contribution of food and beverage groups to total water intake.

Of the total water intake in a day, approximately 51% came from beverages (water, milk, liquid yogurt, coffee and cereal drinks, teas and infusions, alcoholic beverages, and other beverages such as soft drinks, fruit juices, and other commercial beverages with or without sugar/sweetened).Regarding the groups that contributed the most to TWI (Figure 1), water contributed 23% followed by soup (17%) and the “other foods” group (16%).

The groups that contributed with the lowest percentage to TWI were vegetables on the plate (2%), alcoholic beverages (3%) and “other drinks” (3%).

## 5. Discussion

To our knowledge, this is the first study on hydration status using urinary biomarkers in older adults with NCD and showed that one third of the participants had an inadequate hydration status. Elderly people with NCD usually have more comorbidities and a greater dependence on activities of daily living due to perceptual, sensory, and motor skill changes [47,48,49,50,51,52]. Those specific characteristics may include important behavioral changes in the most severe forms of the disease, such as the total loss of self-feeding and hydration capacity. As the disease progresses, dependence and the need for a caregiver also increase [53,54,55,56]. Due to these characteristics, the difficulty in collecting 24-h urine samples is further increased, reinforcing the added value of the results that this study provides.

The best method to assess hydration status has been discussed in the literature. There is scientific evidence that directly measured serum osmolality is the reference standard for water-loss dehydration in older people because it is not affected by failing renal function (common in older people) and directly measures the amount of effective solute in serum or plasma. These characteristics make directly measured serum or plasma osmolality the clear reference standard for the assessment of hydration in older people [23]. Most of the studies use serum osmolality as a reference method and consider, for current or imminent dehydration, an osmolality of 295 to 300 mOsm/kg, and for current or imminent dehydration, an osmolality > 300 mOsm/kg [23,24].

Plasma osmolality is considered a sensitive biomarker of acute dehydration; however, the values seem to be maintained within a narrow range even with large fluctuations in daily volumes of fluid intake [3].

In contrast, renal excretions vary within a wider range of values and according to water losses. The regulation and maintenance mechanisms of hydro-electrolyte balance are quite complex, but briefly, when water intake does not suppress that which is lost, there is a decrease in total body water. Consequently, the production of anti-diuretic hormone increases, which acts by retaining water in the body and making the urine produced more concentrated (increasing urinary osmolality). Thus, higher urinary osmolalities reflect worse hydration status [3,38].

In this study, we used the combination of two urinary methods to classify hydration status: urinary osmolality higher than 500 mOsm/Kg, according to EFSA [28], or a negative FWR [38].

Two studies that evaluated the hydration status of Portuguese older adults without NCD, using the FWR method, the first in a representative sample and the other in a sample of physically active older population living in the community, showed that 16.3% and 8.1% were classified at risk of hypohydration/hypohydration, respectively [1,57]. The FWR values found in the present study were approximately half those found in the first study and were close to those found in the second study. In the second study, the mean urinary osmolality reported was 403 mOsm/Kg in women and 454 mOsm/Kg in men, values close to those we are presenting (438 mOsm/Kg).

None of these studies mentioned the season of urine collection, and, in our study, 70% of urine samples were collected in autumn. Palmisano et al. reported that, during the summer, older adults are more susceptible to dehydration [1]. The same was reported in women by Gamba et al. [8]. Therefore, it can be speculated that the prevalence of hydration inadequacy in our population could be higher if samples had been collected in the summer, when more water is lost through transpiration and may not be adequately compensated by dietary intake.

In our sample, the average contribution of water from beverages represented approximately half of TWI, ranging between 34% and 79%, values that are rather different from those presented in the literature since a contribution of around 80% of water from beverages is reported [58]. These values are, however, in line with those reported by Gonçalves et al. in a Portuguese sample of older adults [1], suggesting that these values are due to cultural or age factors. Several factors can influence the choice of drink and food, such as availability, climate, cultural and religious factors, health status, economic conditions, and age. Consequently, the amount of TWI from beverages and food varies between individuals and countries or settings [11,59].

In relation to water as a beverage, the main contributor to the TWI in this study, it was observed that water intake occurred mostly during meals (lunch and dinner) and when taking medication.

We did not find any significant relationship between medication intake and total water intake, nor did we find any relationship between medication and drinking water. On the contrary, Jimoh et al. [60] discovered that those who drank enough drank more with medication among the elderly living in long-term care. In our study, all elderly people, institutionalized or not, took medication. The average number of medications taken was higher in institutionalized elderly individuals, however, without statistical significance. The low sample size of our study may be a possible justification for the lack of significant differences.

The remaining beverages were taken with a snack, either in the morning or in the afternoon, and at breakfast. Alcoholic beverages were consumed at lunch and/or dinner, as well as other beverages: soft drinks, fruit juices, and other commercial beverages with or without sugar or sweetener. Coffee and cereal drinks, teas and infusions and milk and liquid yogurt were present at breakfast and snacks. The timing and choice of beverages are related to cultural habits. Alcoholic drinks and a group of other drinks replaced the water consumed at lunch and dinner. A TWI of approximately 2000 mL was reported by Gonçalves et al., in line with our results, as well as the main water sources, since in the later study, water and the group “other foods”, composed of foods with low water content, were the main contributors to TWI. In order, the two groups with the lowest contributions were the alcoholic beverages group and the “other beverages” group. The stratification of the sample by sex and the food groups created to assess water sources constituted methodological differences between the studies. We chose to create a group for “vegetables on the plate”, separate from the “soup” group. Interestingly, soup was the second-largest contributor to TWI, and vegetables on the plate were the smallest contributor [1]. In fact, vegetable soup is an important symbol of Portuguese gastronomy, present at lunch and dinner. According to data from the most recent National Survey of Food and Physical Activity, older adults were the second age group that consumed more soup daily. This unit of various foods, usually vegetables, is incorporated into the Portuguese diet from an early age and is usually the first to be introduced in complementary foods. This context may help to explain the fact that soup is, after water, the food that most contributes to TWI [61].

No significant statistical differences were found in the TWI mean according to the median of urinary osmolality groups. Zang et al. explored the relationship between hydration status and TWI in young Chinese adults without chronic diseases and reported that participants with higher TWI had better hydration status with lower urinary osmolality [62]. The methods used to assess the TWI were different from ours, as well as the age sample, which may explain the lack of association since, in the case of older adults, there is a decrease in the capacity of renal concentration and, consequently, this population may need more water to excrete the same load of solutes compared to young adults. The low sample size could also be a possible explanation for the differences observed since the individuals with a urine osmolality equal to or lower than the median presented a higher TWI mean even though there was no statistical significance [58,63].

Unlike Gamba et al., who found that excessive sodium excretion was associated with an increased risk of hypohydration in men, suggesting that sodium intake may not be offset by the intake of water needed to excrete it [8], no significant statistical association was observed between excessive sodium excretion and hydration status in the present study.

The urinary biomarkers used in this study to assess hydration status are considered the most responsive to differences in fluid volume intake since they represent the sum of behavioral and neuroendocrine responses that influence renal concentration and dilution during the day, in accordance with the changes in the TBW. Due to these characteristics, it was suggested as the most suitable for determining individual TWI needs; thus, its use to assess hydration status is considered a strength of the present study [3].

A point that deserves discussion is the fact that the use of urinary osmolality in older adults can cause false states of adequate hydration, insofar as the urinary osmolality can present low values due to the decrease in the renal concentration capacity [46]. This potential limitation was overcome by the concomitant use of the FWR method. This method takes into account the decrease in renal concentrating capacity according to age [38].

Furthermore, sensitivity analyses were performed. There were no statistically significant differences in hydration status when renal patients were excluded from the study.

Unfortunately, it was only possible to collect a single 24-h urine sample to assess hydration status as well as sodium and potassium excretion, which may not be representative of the individual’s usual behavior. However, the collection of one 24-h urine sample was indeed very valuable given the difficulty of 24-h urine collection among the study’s population.

On non-consecutive days, two 24-h food recall questionnaires were used, allowing the collection of information on water sources and TWI. The use of two questionnaires is a strength of this study since it allows a closer approximation to the usual intake than the use of a single recall. Although this method relies on memory, this potential limitation was overcome by the caregiver collaboration in the case of participants being unable to respond.

The sample used may not be representative of older adults with NCD, and the sample size was small, which may limit the results obtained. In addition, clinical data were self-reported, and participants with renal pathology or taking antihypertensive medication were not excluded, although these conditions may influence urinary excretion. As for antihypertensive medication, the type was not specified. It was not possible to assess renal function, for example, through the glomerular filtration rate, in patients with renal disease. These aspects would be important to include in future studies.

## 6. Conclusions

Older adults with NCD are a population group at greater risk of dehydration, and the consequences of hypohydration may even worsen the disease [2,58,64]. Therefore, it is essential not to neglect hydration in this vulnerable population, and it is important to outline strategies for nutritionally adequate water and food intake, reinforcing the need to comply with the recommendations for the TWI. At the same time, encouraging a reduction in sodium intake and an increase in potassium intake is imperative.

Although the water sources that most contribute to TWI are two nutritionally adequate food groups, improvements can be made by guiding this population towards the daily inclusion of foods that are nutritionally rich in water and micronutrients, such as vegetables, dairy products, and fruit. It is important to reinforce the intake of other drinks other than water that do not contain added sugar but are equally palatable, such as teas, infusions, flavoring waters, and cereal drinks.

## Figures and Tables

**Table 1:** Food and beverage groups created to estimate the contribution of food groups to total water intake.

Beverage/Food Groups	Food Included
Water	Bottled water (with or without gas) and tap water.
Teas and infusions	Teas and infusions.
Coffee and cereal beverages	Coffee and cereal beverages.
Other drinks	Soft drinks (carbonated or non-carbonated), fruit juices, and other commercial beverages with or without sugar or sweetener.
Alcoholic beverages	Wine, beer, and spirits.
Soup	All Soups of all kinds with vegetables (with or without potatoes).
Vegetables	Vegetables (excluding the ones in the soup): raw, cooked, canned, and frozen.
Fruits	Fresh fruit.
Dairy	Milk and yogurt.
Other foods	Meat, fish, eggs, pasta, rice, potatoes (excluding potato soup), pulses, breakfast cereals, cheeses, pastries (cakes, cookies, and jams), sugar and fat added, and gelatin.

**Table 2:** Characteristics of the participants according to the median urinary osmolality.

Characteristics of the Participants			Urinary Osmolality (mOsm/Kg)		
		Sample	≤437.5	>437.5	*p*
Sex, n (%)	Female	20 (66.7)	11 (55.0)	9 (45.0)	0.7
Age (years), mean (SD)		76.4 (7.3)	78.9 (5.3)	73.9 (8.2)	0.06
Education level, n (%)	No formal education	9 (32.2)	6 (66.7)	3 (33.3)	0.3
	Formal education	19 (67.8)	7 (36.8)	12 (63.2)	
Marital status, n (%)	Married/civil union	19 (63.3)	7 (36.8)	12 (63.2)	0.1
	Single/Widow	11 (36.7)	8 (72.7)	3 (27.3)	
Living situation, n (%)	Family member	23 (76.7)	10 (43.5)	13 (56.5)	0.4
	Living in residential units/nursing homes	6 (20)	4 (66.7)	2 (33.3)	
	Alone	1 (3.3)	1 (100)	0 (0)	
Aetiological subtypes, n (%)	Mild NCD	4 (14.8)	2 (50)	2 (50)	0.9
	Major NCD due to Alzheimer’s disease	5 (18.5)	2 (40)	3 (60)	
	Major NCD due to vascular disease	3 (11.1)	1 (33.3)	2 (66.7)	
	Major NCD due to Multiple aetiologies	5 (18.5)	2 (40)	3 (60)	
	Major NCD due to unspecified condition	9 (33.3)	6 (66.7)	3 (33.3)	
	NCD due to substance or medication-induced	1 (3.7)	0 (0)	1 (100)	
Number of comorbidities, mean (SD)		4.0 (2.2)	4.5 (2.4)	3.7 (1.9)	0.3
Number of drugs intake, mean (SD)		8 (3.4)	8.3 (2.8)	7.7 (4)	0.6
Severity of major NCD, n (%)	Questionable NCD–Mild NCD	16 (53.3)	8 (50)	8 (50)	1
	Moderate NCD–Severe NCD	14 (46.7)	7 (50)	7 (50)	
Sodium excretion, n (%)	Excessive	21(70)	10 (47.6)	11 (52.4)	1
Potassium excretion, n (%)	Insufficient	29(96.7)	15 (51.7)	14 (48.3)	1
Na/K ratio excretion, n (%)	Excessive	28(93.3)	14 (50)	14 (50)	1
Body mass index, n (%)	Under weight–Normal weight	6 (20.6)	1 (16.7)	5 (83.3)	0.2
	Overweight–Obese	22 (79.4)	13 (59.1)	9 (11)	
Waist circumference, n (%)	No risk	7 (26)	2 (50)	2 (50)	1
	Hight risk–Very High risk	20 (74)	11 (52.4)	10 (47.6)	
Waist-Hip ratio, n (%)	No risk increase	4 (16.0)	2 (28.6)	5 (71.4)	0.4
	Substantially increased risk	21 (84.0)	11 (55.0)	9 (45.0)	
Fat-free mass %, mean (SD)		39.26 (6.4)	40.5 (6.7)	38 (6.2)	0.3
Fat mass %, mean (SD)		39.50 (8)	40.9 (8.3)	38.1 (7.8)	0.4
Instrumental activities of daily living, n (%)	Independent	20 (3.4%)	8 (53.3%)	12 (85.7%)	0.1
	Slightly dependent	6 (20.7%)	5 (33.3%)	1 (7.1%)	
	Moderately dependent	2 (6.9%)	1 (6.7%)	1 (7.1%)	
	Severely dependent	1 (3.4%)	1 (6.7%)	0 (0%)	
Physical activity *, median (P25; P75)		3.25 (2.5; 4.3)	3.25 (2.5; 3.6)	3.13 (2.7; 4.3)	0.8
Quality of life, mean (SD)		30.4 (4.5)	29.3 (5.2)	31.5 (3.5)	0.2

SD: Standard deviation; P25: percentile 25; P75: percentile 75; NCD: neurocognitive disorder. Excessive sodium excretion was defined as ≥2000 mg/day. Insufficient potassium excretion was considered when <3510 mg/day and the Na/K excess if greater than 1, according to the World Health Organization cut-off points. For the waist circumference, the categories were: no risk (women: <80 cm; men: <94 cm); high risk (women: ≥80 cm and ≤88 cm; men: ≥94 cm and ≤102 cm); and very high risk (women: >88 cm; men: >102 cm) Due to the small number of participants in each category, we opted to combine the categories “high risk” and “very high risk” into one category. For the waist-hip ratio the categories were: no risk increased for substantial metabolic (women < 0.85 cm and men < 0.90 cm) and increased risk for substantial metabolic (women ≥ 0.85 cm and men ≥ 0.90 cm). Independence in performing instrumental activities of daily living was assessed using the Barthel Index. *: The distribution of this variable in the sample was different from the normal distribution, and in this case the Mann’s test—Whitney was used.

**Table 3:** Contribution of water from beverages and food and dietary intake according to the median of urinary osmolality.

Contribution of Water from Beverages and Food		Urinary Osmolality (mOsm/Kg)		
		≤437.5	>437.5	*p*
Water, mean (SD)	%	24.4 (13.1)	21.7 (11.7)	0.6
Soup, mean (SD)		19.0 (10.5)	15.4 (11.4)	0.4
Others foods, mean (SD)		15.0 (6.8)	16.2 (5.4)	0.8
Dairy, mean (SD)		13.7 (10.3)	15.3 (13.2)	0.7
Fruits, mean (SD)		13.3 (7.5)	13.7 (10.6)	0.9
Teas and infusions *, median (P25, 75)		0 (0; 11.2)	0 (0; 11.1)	0.9
Coffee and cereal beverages *, median (P25, 75)		3.8 (0.1; 8.2)	2.0 (0.1; 5.5)	0.5
Alcoholic beverages *, median (P25, 75)		0 (0; 0)	0 (0; 5)	0.2
Other drinks *, median (P25, 75)		0 (0; 3.8)	0 (0; 4.4)	0.7
Vegetables on the plate *, median (P25, 75)		0.5 (0; 2.8)	2.4 (0.9; 3.7)	0.1
Dietary intake				
Total water intake, mean (SD)	mL	2076 (950)	1947 (737)	0.7
Energy, mean (SD)	Kcal	1607 (444)	1806 (568)	0.3
Protein *, median (P25; P75)	G	60.8 (45.5; 73.2)	66.9 (56.2; 86.1)	0.1
Fat, mean (SD)		40.4 (14.5)	46.4 (15.3)	0.3
SFA, mean (SD)		10.4 (5)	13 (6.9)	0.3
Carbohydrates, mean (SD)		213.1 (65.4)	252.8 (94.6)	0.2
Protein *, median (P25; P75)	%TEI	15.1 (12.6; 18)	16.2 (14.4; 18)	0.3
Fat, mean (SD)		22.9 (6.2)	23.6 (5.6)	0.8
SFA, mean (SD)		5.8 (2.3)	6.6 (3.1)	0.5
Carbohydrates, mean (SD)		52.9 (7.7)	55.6 (11.4)	0.5

SD: Standard deviation; P25: percentile 25; P75: percentile 75; TEI: total energy intake Total water intake is equivalent to the water content of food and beverages. *: The distribution of this variable in the sample was different from the normal distribution, and in this case the Mann’s test—Whitney was used.

## Data Availability

Not applicable.

## Appendix A

**Table A1:** Characteristics of the participants according to the severity of major NCD.

Characteristics of the Participants			Severity of Major NCD, n (%)		
		Sample	Questionable NCD–Mild NCD	Moderate NCD–Severe NCD	*p*
Sex, n (%)	Female	20 (66.7)	11 (55%)	9 (45%)	1
Age (years), mean (SD)		76.4 (7.3)	75.4 (6.4)	77.5 (8.3)	0.4
Education level, n (%)	No formal education	9 (32.2)	5 (55.6%)	4 (44.4%)	1
	Formal education	19 (67.8)	11 (57.9%)	8 (42.1%)	
Marital status, n (%)	Married/civil union	19 (63.3)	8 (42.1%)	11 (57.9%)	0.1
	Single/Widow	11 (36.7)	8 (72.7%)	3 (22.3%)	
Living situation, n (%)	Family member	23 (76.7)	10 (43.5%)	13 (55.6%)	0.1
	Living in residential units/nursing homes	6 (20)	5 (83.3%)	1 (16.7%)	
	Alone	1 (3.3)	1 (100%)	0 (0%)	
Aetiological subtypes, n (%)	Mild NCD	4 (14.8)	n.a	n.a	0.6
	Major NCD due to Alzheimer’s disease	5 (18.5)	2 (40%)	3 (60%)	
	Major NCD due to vascular disease	3 (11.1)	3 (100%)	0 (0%)	
	Major NCD due to multiple aetiologies	5 (18.5)	3 (60%)	2 (40%)	
	Major NCD due to unspecified condition	9 (33.3)	5 (55.6%)	4 (44.4%)	
	Major NCD due to substance/medication-induced	1 (3.7)	0 (0%)	1 (100%)	
Number of comorbidities, mean (SD)		4.0 (2.2)	4.8 (2.3)	3 (1.2)	**0.02**
Number of drugs intake, mean (SD)		8 (3.4)	8.8 (2.3)	6.8 (4.5)	0.1
Sodium excretion, n (%)	Excessive	21(70)	12 (57.1%)	9 (42.9%)	0.7
Potassium excretion, n (%)	Insufficient	29(96.7)	15 (51.7%)	14 (48.3%)	1
Na/K ratio excretion, n (%)	Excessive	28(93.3)	15 (53.6%)	13 (46.4%)	1
Body mass index, n (%)	Under weight–Normal weight	6 (20.6)	3 (50%)	3 (50%)	1
	Overweight–Obese	22 (79.4)	12 (54.5%)	10 (45.5%)	
Waist circumference, n (%)	No risk	7 (26)	2 (28.6%)	5 (71.4%)	0.4
	Hight risk–Very High risk	20 (74)	11 (55%)	9 (45%)	
Waist-Hip ratio, n (%)	No risk increase	4 (16.0)	2 (55%)	2 (55%)	1
	Substantially increased risk	21 (84.0)	11 (52.4%)	10 (47.6%)	
Fat-free mass %, mean (SD)		39.26 (6.4)	39.8 (7.2)	38.7 (5.7)	0.6
Fat mass %, mean (SD)		39.50 (8)	39.8 (7.2)	38.7 (5.6)	0.8
Instrumental activities of daily living, n (%)	Independent	20 (3.4%)	13 (65%)	7 (35%)	0.2
	Slightly dependent	6 (20.7%)	3 (50%)	3 (50%)	
	Moderately dependent	2 (6.9%)	0 (0%)	2 (100%)	
	Severely dependent	1 (3.4%)	0 (0%)	1 (100%)	
Physical activity *, median (P25; P75)		3.25 (2.5; 4.3)	2.9 (2.5; 4.2)	3.5 (2.6; 4.3)	0.6
Quality of life, mean (SD)		30.4 (4.5)	31.8 (3.7)	28.6 (4.9)	0.1

SD: Standard deviation; P25: percentile 25; P75: percentile 75; NCD: neurocognitive disorder; n.a.: not applicable. Excessive sodium excretion was defined as ≥2000 mg/day. Insufficient potassium excretion was considered when <3510 mg/day and the Na/K excess if greater than 1, according to the World Health Organization cut-off points. For the waist circumference, the categories were: no risk (women: <80 cm; men: <94 cm); high risk (women: ≥80 cm and ≤88 cm; men: ≥94 cm and ≤102 cm); and very high risk (women: >88 cm; men > 102 cm) Due to the small number of participants in each category, we opted to combine the categories “high risk” and “very high risk” into one category. For the waist-hip ratio the categories were: no risk increased for substantial metabolic (women < 0.85 cm and men < 0.90 cm) and increased risk for substantial metabolic (women ≥ 0.85 cm and men ≥ 0.90 cm) Independence in performing instrumental activities of daily living was assessed using the Barthel Index. Bold: Statistically significant. *: The distribution of this variable in the sample was different from the normal distribution, and in this case the Mann’s test—Whitney was used.
